# Grounding grammatical categories: attention bias in hand space influences grammatical congruency judgment of Chinese nominal classifiers

**DOI:** 10.3389/fpsyg.2015.01299

**Published:** 2015-08-27

**Authors:** Marit Lobben, Stefania D’Ascenzo

**Affiliations:** ^1^Department of Psychology, University of Oslo, OsloNorway; ^2^Department of Communication and Economics, University of Modena and Reggio Emilia, Emilia-RomagnaItaly

**Keywords:** grounding, abstract concepts, peripersonal space, endogenous attention, Chinese numeral classifiers

## Abstract

Embodied cognitive theories predict that linguistic conceptual representations are grounded and continually represented in real world, sensorimotor experiences. However, there is an on-going debate on whether this also holds for abstract concepts. Grammar is the archetype of abstract knowledge, and therefore constitutes a test case against embodied theories of language representation. Former studies have largely focussed on lexical-level embodied representations. In the present study we take the *grounding-by-modality* idea a step further by using reaction time (RT) data from the linguistic processing of nominal classifiers in Chinese. We take advantage of an independent body of research, which shows that attention in hand space is biased. Specifically, objects near the hand consistently yield shorter RTs as a function of readiness for action on graspable objects within reaching space, and the same biased attention inhibits attentional disengagement. We predicted that this attention bias would equally apply to the graspable object classifier but not to the big object classifier. Chinese speakers (*N* = 22) judged grammatical congruency of classifier-noun combinations in two conditions: graspable object classifier and big object classifier. We found that RTs for the graspable object classifier were significantly faster in congruent combinations, and significantly slower in incongruent combinations, than the big object classifier. There was no main effect on grammatical violations, but rather an interaction effect of classifier type. Thus, we demonstrate here grammatical category-specific effects pertaining to the **semantic content** and by extension the visual and tactile **modality of acquisition** underlying the acquisition of these categories. We conclude that abstract grammatical categories are subjected to the same mechanisms as general cognitive and neurophysiological processes and may therefore be grounded.

## Introduction

A crucial question in the study of linguistic meaning is whether semantics can be defined in terms of word–word and morpheme–morpheme contextual relations only, or if some additional grounding also play a role that links the linguistic representation to the objects and events of reference (i.e., word-world relations) when humans use and understand language. Although children evidently learn language in a context of direct reference to objects, events and situations, it is less clear how the acquisition process later affects how linguistic representations are stored and retrieved in adulthood. *A priori* it is entirely possible that linguistic knowledge, whose grammatical systems pass on at a relatively slow changing pace through generations, in the individual the result of implicit learning (cf. Escandell-Vidal et al., 2011, implicit learning of artificial grammars in Reber, 1967; Daltrozzo and Conway, 2014; Folia and Petersson, 2014; Schuchard and Thompson, 2014, for grammar in procedural memory see Ullman et al., 1997; Ullman, 2001a,b; Tomblin et al., 2007), fully automatized and non-conscious, attains an autonomous status in self-contained systems once learned; previously it was generally held that language is represented in a separate module in the brain isolate from general cognition and that the human language faculty basically consists of amodal symbol manipulation and grammatical rules (Chomsky, 1957; Fodor, 1980, 1983; Pylyshyn, 1984; Anderson, 1990; Hauser et al., 2002; Jackendoff, 2002). In contrast, later proposals imply that language is a special case of general cognition. Embodiment views of language and cognition respond to *the grounding problem* (Searle, 1980; Harnad, 1990), and are based on the idea that cognitive representations derive from and are shaped by the type of experiences humans have with their bodies. We do not have a direct mind-free access to the external world, but rather conceptualize it through how we experience it in bodily interaction. If language, then, is a special case of general cognition (Langacker, 1987), and not amodal and encapsulated, we can expect that linguistic concepts and categories be grounded in the same way.

If the body constrains how concepts are constructed, the consecutive use of these concepts depends on how these experiences can be successfully retrieved from memory. The crucial question is whether understanding also implies recollecting the *manner in which a concept was acquired*. A basic assumption in grounded theories of cognition is the fact that concepts are fundamentally perceptual in nature. This belief has developed in parallel with the emerging neuroimaging studies and behavioral data (for a review, see Barsalou, 2008) showing that modality-specific perceptual (Kan et al., 2003; Gonzales et al., 2006; Trumpp et al., 2013; Amsel et al., 2014) and motor (Jeannerod, 2001) areas of the brain are recruited when the meaning of concepts and sentences are understood. When conceptual stimuli are processed, perceptual, and motor areas are activated that *correspond to the meanings* of these words; supporting the idea that conceptual processing is built upon sensorimotor processes (Gallese and Lakoff, 2005; Goldberg et al., 2006). The retrieval of conceptual meaning thus involves a partial re-enactment (simulation) of experiences during concept acquisition (Barsalou, 1999, 2008; Barsalou et al., 2003).

The particular grounding features relevant to nominal classifier categories can often be associated with one or more sensory and motor modalities, typically visual, or tactile (Lee, 1987). All types of nominal classifiers, such as the numeral classifiers in Chinese, are examples of grammatical units that generalize over a large set of nouns, taking but a few of the common semantic features of these nouns as its sole semantic content. Diachronically, such morphemes generally derive from a lexical item, and extract a property seen as relevant to other referents that possess the same feature.

Cross-linguistically, as in Chinese, so-called *sortal* classifiers describe the semantic essence of an ontologically, functionally or culturally determined category, typically HUMAN, ANIMAL, FOOD, TOOL, or in relation to cognitively salient features like SIZE, SHAPE, or MATERIAL classes (Aikhenvald, 2000; Grinevald, 2000; Senft, 2000). Hence, semantic or other cognitive aspects of the noun determine the choice of a particular classifier. Chinese has several dozens of classifiers, some more and others less frequently used (for a full list, see, e.g., Chao, 1968, section 7.9). A typical linguistic function of classifiers is reference tracking in communicative situations, narrowing down the scope of potential referents. These deictic elements therefore behave as typical grounding devices, supported by the fact that through time, classifiers commonly develop into pronouns, the ultimate grounding device in language. Chinese, as well as several other Asian languages (e.g., Japanese, Vietnamese), classify nouns inter alia according to the **graspability affordances** (Gibson, 1966, 1977) of the noun referents when these nouns occur with numerals or demonstrative pronouns (i.e., in referencing expressions). This type of classifier semantics provides a yet unexplored link to an independent field of research within cognitive psychology and neuroscience.

Cognitive and neurophysiological processing in peripersonal **hand space** is a well-researched area that lends itself to testing whether a cognitive bias influences linguistic processing. In this field, investigations have shown that while attention to graspable objects in near space engages human attention in preparation for action (Reed et al., 2006), attention is also harder to disengage from objects within hand space (Abrams et al., 2008; Tseng et al., 2012) due to the constant alertness for action toward graspable objects in our immediate vicinity. Since graspable objects in near space enjoy this special status of mental and physical immediacy as well as a type of inertia that causes the mind to linger, grammatical categories referring to graspable objects are apt to investigate if there is a difference in how abstract grammatical categories are mentally integrated and grounded. We therefore wanted to explore the possibility that such effects apply not only to attention, perception and memory of real-world, concrete objects, but also to the semantic processing of non-conscious, implicitly acquired linguistic concepts related to hand and action space. These grammatical elements are also part of the speakers’ established knowledge systems, in other words, top–down knowledge. Crucially, *if grammatical categories are indeed grounded by sensorimotor modality, it follows that any idiosyncrasy of these modalities will affect storage and retrieval of the grammatical categories in the exact same manner as in perception and cognition.* Could top–down, non-conscious processing of grammatical affixes related to the hand be linked to attention processing? In the following, we demonstrate that nominal classifiers are indeed grounded, and likely derived from and continually represented in humans’ sensory and motor experiences in the world.

How does the hand affect cognition? The position of the hand influences our perception of the environment, attentional control, as well as memory and higher-order cognition (Brockmole et al., 2013). Moreover, sensory and attentional networks appear to be interlinked. In patients with visual neglect (heminanopsia), the ability to detect objects in the impaired hemifield can be nearly doubled if the patient’s hand is placed in the blind field (Schendel and Robertson, 2004). Placing the hand near an object can also affect early preattentive processes in healthy subjects, e.g., the segregation of objects from backgrounds (Cosman and Vecera, 2010). Brockmole et al. (2013) explain these perceptual enhancements as a potential ‘shift from the perception-oriented parvocellular pathway toward the action-oriented magnocellular pathway’ when objects appear near the hands, an assumption that is consistent with recent works (Gozli et al., 2012; Goodhew et al., 2013; Weidler and Abrams, 2014).

Two types of **attention bias** have been demonstrated in hand space: faster engagement and slower disengagement. For what concerns faster engagement, Reed et al. (2006, 2007, 2010) developed an *embodied model of spatial cognition* through several studies (Garza et al., 2013), which investigated the effect of hand, trunk, head direction and body posture on attention, arguing that early, sensory-related processing in the body has an effect on visual processing. This fostered a surge of studies on biased attention in tool and hand space (e.g., Tipper et al., 1998; Handy et al., 2005; Meinert, 2011; Gozli et al., 2012; Tseng et al., 2012; Goodhew et al., 2013; Langerak et al., 2013; Thomas, 2013). Reed et al. (2010) found that targets appearing in the hand’s grasping space – near the palm – *produced faster responses* than when the targets were presented to the back of the hand or the forearm. The same effect was observed using a rake (i.e., a tool). They point out that bottom–up modulation of attention can be modulated on the basis of inputs from the body as well as from visual cues, and that changing the body position can change the salience of space. Hence, a likely effect of peripersonal space is a readiness to act within that space, and that this readiness, in addition to being supported by special visuotactile cells, is aided by endogenous, or top–down attention to objects in an individual’s immediate vicinity.

Contrary to this, with respect to slower disengagement, it has been shown that items near the hand demand longer processing times than comparable situations away from the hand. For instance, Abrams et al. (2008) reported a difficulty of disengagement effect for objects in hand space. The authors demonstrated that people were slower to disengage their attention from the visual stimuli in both space (inhibition of return and visual search experiments) and time (attentional blink task) when these were presented near the hand. In addition, a difficulty-of-disengagement effect was also observed in a top–down conscious processing study by Davoli and Abrams (2009), who found in a visual search task that participants searched through the display at a slower rate when they held their hands behind their backs and imagined their hands to be near the display.

Thus, attention bias in hand space has been demonstrated for perception and cognition, but does it also parallel grammatical processing of abstract semantic categories? In the grounding debate, there exists some confusion on what makes a concept abstract. A clarification with respect to the nature of the stimuli used in this experiment is therefore in place. A primary characteristic of grammatical categories is their general applicability, responding to the diverse situations in our everyday lives: a minimum of semantic features makes the category maximally versatile. An effect of this flexibility is that it distances itself from the individual concrete objects and situations. Therefore, the degree to which a morpheme is **grammaticalized** reflects its abstraction level. Grammaticalization is the diachronic process in which a lexical item changes to a grammatical item while its meaning also changes from more concrete/specific to more abstract/general (Zhiqun Xing, 2012). Highly grammaticalized morphemes are more abstract in the sense that they are semantically more general, more text-frequent and syntactically defined, as well as phonologically simpler than its historical source than comparable morphemes in less grammaticalized languages (Bybee, 2003; Narrog and Heine, 2011). Nominal classifiers in Chinese have in general been subject to grammaticalization processes during the last two 1000 plus years (Erbaugh, 1986), turning once lexical items into abstract grammatical morphemes. For example, the original meaning of the default classifier gè 个, which today is devoid of meaning and serves only to fulfill a grammatical requirement, was ‘bamboo’. To what extent are classifiers used in this experiment grammaticalized? Although traditionally classified as an isolating language, Chinese demonstrates a consistently deeper level of grammaticalization than for example Thai, a language with parallel constructions (Post, 2007). In these parallel constructions, the Chinese forms exhibit greater degrees of semantic bleaching, syntactic behavioral restrictions, and phonological erosion. With respect to the classifiers used as stimuli in the present investigation, several conditions confirm their grammaticalized status. First, these nominal classifiers can be syntactically defined since they are linguistic morphemes mandatorily occurring when a number or a deictic element or measure term is part of the noun phrase, e.g., with *yí* ‘one’, *san* ‘three’, *shí* ‘ten’, with the quantifying expression *bàn* ‘half’ and with demonstrative pronouns, e.g., *zhèi* ‘this’, *nèi* ‘that’, or *nìi* ‘which’. The structural status of classifiers is also evident by the fact that Chinese uses a default classifier *gè* (个) whenever a special classifier is not appropriate, because the slot in the noun phrase has to be filled for the phrase to be grammatical. The use of the bǎ (把) classifier is evidently highly *productive*, demonstrating its involvement in a grammatical rule^1^. A canonical trait of grammaticalization is that the process unfolds across several generations of language learners, and therefore evolves at the level of implicit learning and non-conscious processing. Once a source lexeme has given rise to one or several novel usages, it may eventually get lost or replaced by near equivalents, leaving speakers no option to backtrack semantics. The established grammatical morphemes will then be stored as abstract concepts with relevance to grammar only. The classifier for graspable objects displays such traits of grammaticalization. Its original function was lexical: in Middle Chinese, it functioned as a verb meaning ‘to grasp, to take hold of’ (Peyraube, 1996). In modern Mandarin Chinese, however, ba (unmarked for tone in this paragraph on its historical origin) is no longer used as a main verb or noun, since these have now been replaced by compounds (Post, 2007). *Ba* has grammaticalized into three *synchronically unrelated usages*. From the verbal function three grammatical functions emerged: (1) an accusative marker in transitive sentences (Chao, 1968; Thompson, 1973; Li and Thompson, 1974; Liu, 2013; in Post, 2007 referred to as the ‘manipulated object construction’), (2) a purposive marker, and (3) a nominal classifier used to classify nouns with numerals or demonstrative pronouns. The original ‘grasp’ meaning of the lexical verb has been lost and replaced another verb *ná* ‘to take’. Hence, the usage of *ba* as a lexical verb no longer is considered grammatical, cf. tâ zhi xi tâ ng ná (*bâ) yî-ge lí [3p only reckon take (*take) one-CL pear] ‘He only planned to take a single pear.’ By comparison, the equivalent ’aw_Manipulated.Obj.Marker_ in Thai has retained its status as a full verb (Post, 2007). In addition, tonal loss of *ba* in discourse is observed with some speakers of Chinese, possibly pointing to a first stage of phonological erosion (Post, 2007). The above serves to illustrate that these present-day usages of *ba* clearly fulfill **highly specialized grammatical functions**. Post (2007) concludes that ‘[b]oth the increasingly abstract semantics and the high degree of event-integration in ba-marked clauses, support a view of relatively advanced grammaticalization.’

With respect to the other classifier used in this experiment, zuò 座, for big, stationary objects, its semantics is related to the word for ‘to sit’ 座. Part of its semantics is that these referents, in addition to being non-movable objects, would be too large to physically hold in hand, and this classifier is therefore a useful contrast and control to the special semantics associated with the graspable noun classifier.

In the current study we aimed at investigating the abstract grammatical representation of haptic affordances contrasted with a comparable grammatical category that did not imply such affordances in Mandarin Chinese, to look for potential grounding effects in the former. Specifically, we tested whether the nominal classifier bǎ, which is used in conjunction with nouns denoting graspable objects, is processed differently from the classifier zuò, which is used with nouns that refer to big and stationary items that cannot be handheld. We only included senses of the graspable-object classifier in which it directly signifies object affordances, and not in its less common usage as ‘a handful’, in which case it rather functions as a container classifier. This separate type of quantifier construction is semantically distinguishable since it occurs only with mass nouns. This usage aligns with many other body part containers, e.g., yi liǎn huî [one face dust] ‘a faceful of dust,’ yi dùzi qì [one stomach anger] ‘a stomachful of grievance,’ and yi tóu bái fâ [one head white hair] ‘a headful of white hair’ (Li and Thompson, 1981). No such instances of bǎ were included in the stimuli. Since the RTs were measured at the appearance of the noun, the two semantic types cannot be confused.

We predicted that the classifier categorizing graspable object nouns would prime toward *engaging Chinese speakers’ attention* based on experiences of preparation for action; likewise that this classifier would prime toward a more arduous *disengagement of attention* than the big object nominal classifier when the classifier-noun pair was incongruent (ungrammatical). Consequently, a readiness for action-effect should not only predict faster RTs in the graspable classifier condition when the classifier-noun pairs are congruent (grammatical), but also show a concomitant slower disengagement-effect when the classifier-noun pairs are incongruent.

We also predicted an interaction effect for classifiers, specifically that the congruent classifier-noun pairs in the graspable classifier condition would be associated with faster grammaticality judgments compared to congruent classifier-noun pairs in the big classifier condition. Likewise, we predicted that incongruent classifier-noun pairs in the graspable classifier condition would be associated with longer RTs compared to incongruent classifier-noun pairs in the big classifier condition. Here, we expected that the recognition of a non-graspable noun that violate grammar to be significantly delayed, in which case the priming of the graspable classifier should result in a difficulty of disengagement effect. The participants’ recognition of congruent graspable nouns, by contrast, would be facilitated by the already alerted attention, due to the classifier’s priming effect. That is, RTs will be significantly longer than in the control condition when the classifier-noun pair is incongruent than when it is congruent.

If this pattern is confirmed, there will be reason to think that the graspable object classifier is grounded. Conversely, if there is no significant difference in reaction times (RTs) between the congruent big and grasp classifier conditions, this will suggest that the two classifiers are stored and processed in the same manner, in other words, an argument in favor of amodal theories. In that case, there is no reason to think that what is known about attention in peripersonal space in the neurophysiological or behavioral paradigms has any consequences for how language is stored and processed. Another possible outcome is that there will be a difference between congruent and incongruent trials such that incongruent trials will be processed slower than both congruent conditions. In that case, the main effect is on congruency and attributable to a general grammatical violation effect. This RT pattern will also corroborate amodal representation of nominal classifiers.

## Experiment

### Method

#### Participants

Twenty-five native Chinese speakers (Mean age = 28.5; SD = 4.4; 13 females) took part in the experiment. They were recruited from an internet-based forum for Chinese students residing in Norway. All participants reported being right-handed. All had normal, or corrected-to-normal visual acuity, and normal hearing. All procedures conformed to national and institutional guidelines and the Declaration of Helsinki. Participants gave their written informed consent before taking part in the study, and were compensated NOK 500 at the end of the session.

#### Design and Materials

Participants performed a grammaticality judgment test that involved correct and incorrect combinations of two SIZE DOMAIN classifiers (CL): 把 bǎ, used for small, graspable objects (typically kitchen and agricultural tools and other objects with a handle such as umbrellas, teapots, abaci; musical instruments with a neck: cello, violin, and other hand-size objects), and the classifier 座 zuò, which is used for large, stationary objects (typically large constructions like bridges, mansions and skyscrapers, public parks, icebergs and mountains). The task was to decide whether Chinese classifier-noun pairs are grammatical (congruent) or ungrammatical (incongruent). The stimuli was in part drawn from the MDBG online Chinese dictionary, and in part from the Chinese equivalent of Google, baidu.com, by searching for combinations of the Chinese number ‘one’ in conjunction with each classifier. Five native Chinese speakers subsequently controlled that congruent classifier-noun pairs were indeed grammatical, and that incongruent pairs were ungrammatical. In the first condition, a typical congruent trial is the numeral noun phrase 一把…手枪 [Yî bǎ … shǒuqiāng] ‘one- CL:GRASPABLE.OBJECT...pistol’, and in the incongruent trial 一把…山脉 [Yî bǎ … shān mài] ‘one- CL:GRASPABLE.OBJECT … mountain range.’ In the second condition for the big object classifier, a typical congruent noun phrase is 一座…桥 [Yî zuò... qiáo] ‘one-CL:BIG.OBJECT bridge’ and incongruent 一座…葡萄 [Yî zuò... pútao] ‘one-CL:BIG.OBJECT grape’. A full list of the stimuli is provided in the supplemental material.

#### Procedure

The participants performed a practice session prior to the experiment to prevent effects related to inter-individual differences in task learning. The experiment was divided into two sessions, each session lasting for ~C;15 min. During the short break between the first and second session, participants were instructed to rest.

The design was an orthogonal combination of two experimental within factors: 2 Congruency (*congruent, incongruent*) × 2 Classifiers (*graspable, big*). Thus, there were four conditions of trials, (1) graspable-congruent, (2) graspable-incongruent, (3) big-congruent, and (4) big-incongruent. 40 grammatically correct pairs were used in each congruent condition, and 20 grammatically incorrect pairs were used in each incongruent condition. The classifier-noun combinations were compound trials, i.e., trials consisting of two separate stimuli. Compound trials required a yes-no response. In addition, participants were presented with 20 partial trials (Ollinger et al., 2001a,b) and 44 randomized short periods of rest, each equal in length to one trial. Partial trials presented only the classifier followed by a blank screen in the noun time window, and did not require a grammaticality judgment on part of the participants. These were therefore not included in the RT calculations. Altogether, each participant received all the 120 pairs of congruent and incongruent combinations, following proper across trial counterbalancing.

**FIGURE 1:**
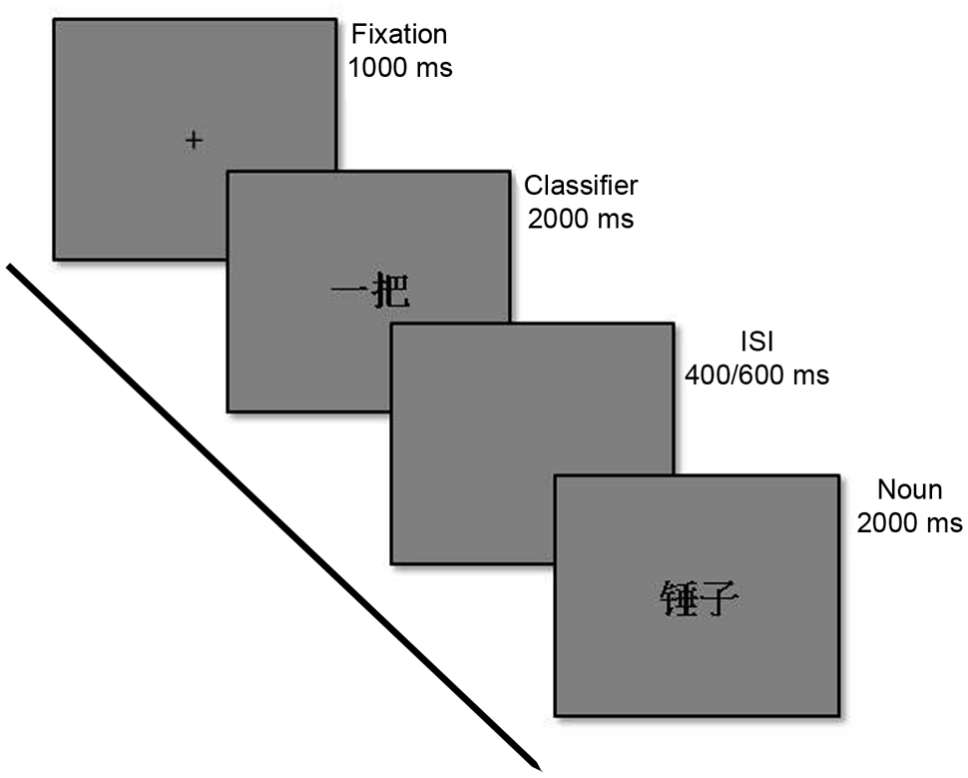
**Sequence of events in a representative trial of the congruent graspable objects classifier condition**.

The RT data was collected in an fMRI experiment while participants were also scanned (Philips Achieva 3 Tesla MR scanner, Philips Medial Systems, Netherlands). The image data are not reported in this article. The experiment was implemented in E-Prime 2.0 (Psychological Software Tools, Inc., Pittsburgh, PA, USA), and presented on an MR-compatible LCD-screen (NNL LCD Monitor^®^, NordicNeuroLab, Bergen, Norway), placed at the head of the scanner bore, and projected to the supine participants via a mirror mounted on the coil that was positioned over the participants’ heads. Screen resolution was set to 1920 × 1080 at 60 Hz, and field of view measured 32° visual angle, effective viewing distance 1.2 m. All characters were presented in the font “SimSun”, font size 58. All stimulus displays were made equal in terms of luminance and visual complexity. In each trial, participants were first presented a fixation display consisting of a black (RGB: 0, 0, 0) centered crosshair on a gray (RGB: 128, 128, 128) background. Participants were instructed to fixate the crosshair from fixation display onset and until display offset. After 1000 ms the fixation display was replaced by a classifier display consisting of a Chinese numeral-classifier (color black) on a gray background for 2000 ms, followed by a short inter-stimulus interval (ISI) displaying an empty gray display for either 400, or 600 ms. ISI duration was fully randomized and could thus not be predicted in advance. The noun display followed immediately after ISI display offset. In this display, the participants were presented a Chinese noun (color black) on a gray background for 2000 ms. Both classifiers and nouns were fully randomized in order to prevent the participant from adapting to a response pattern due to regularities in the program. Participants were instructed to give their responses by pressing the right index finger-button for correct pairs, and the right middle finger-button for incorrect pairs on the response pad. The response had to be given before noun display offset. Any responses occurring outside this time frame were not logged, and these trials were excluded from data analysis. In addition, incorrect responses, rest trials and partial trials were excluded from the analysis (44% of total trials) leaving 2095 trials to analyse. A gray inter-trial interval (ITI) display lasting 1000, 5000, or 9000 ms was presented between each experiment trial, and the duration of the ITI displays were fully randomized (see **Figure 1** for the sequence of events over a trial).

#### Data Analysis

Both accuracy and RTs were recorded for each of the four trial types. The percentage of correct responses was calculated to measure each participant’s performance accuracy. Two participants out of 24 were excluded from the analyses because overall accuracy failed to reach 90%. Finally, 22 participants were included in the behavioral analyses. Incorrect responses (4.13% of trials) or latencies that were 2 standard deviation above or below each participant’s mean (5.15% of trials) were excluded from the analyses.

Within-subject analyses were performed on RTs (ms) by means of repeated-measures ANOVA using *Congruency* (congruent, incongruent) and *Classifier* (graspable, big) as within-subjects factors. A post-hoc analysis using the Duncan test was used for multiple comparisons. The level of statistical significance was set at 5% (*p* < 0.05).

## Results

**FIGURE 2:**
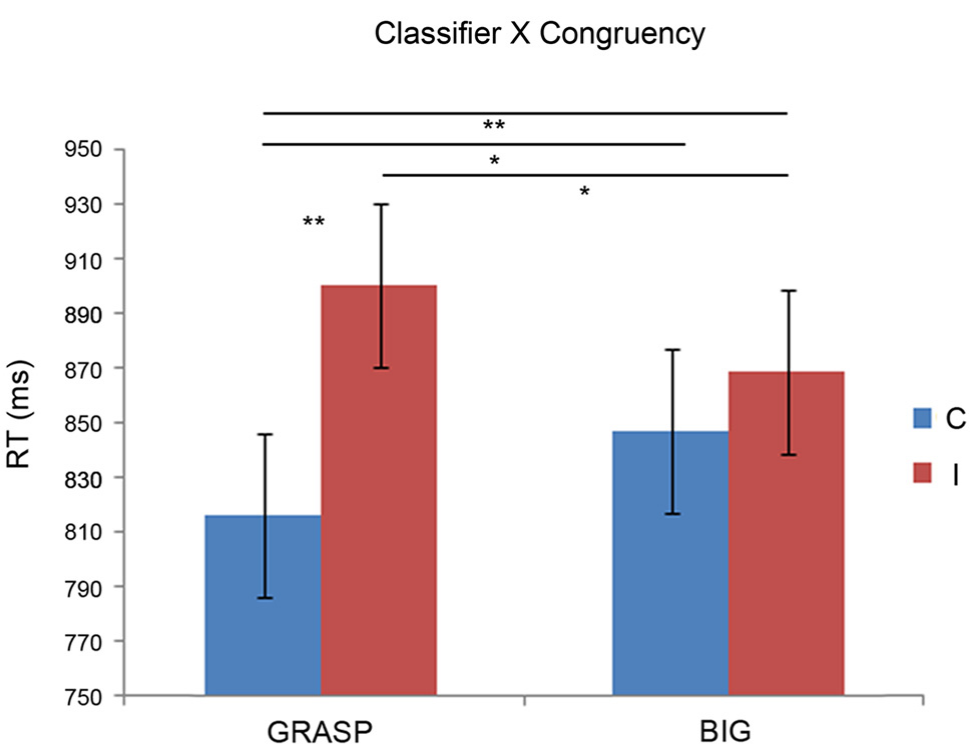
**Mean reaction times (RTs) as a function of Classifiers and Congruency**. Error bars represent 95% confidence intervals computed with the formula of Loftus and Masson (1994) for within-subject designs. Asterisks denote significant *post hoc* comparisons: *p < 0.05; **p < 0.005. C, congruent; I, incongruent.

The ANOVA showed significant main effect of Congruency, *F*_(1,21)_ = 12.67, *p* < 0.005, η^2^ = 0.924. Participants were faster in the congruent condition (*M* = 831.8) than in the incongruent condition (*M* = 884.82). Furthermore, results demonstrated a significant interaction between Classifier × Congruency, *F*_(1,21)_ = 8.72, *p* < 0.01, η^2^ = 0.804. In the graspable congruent (*M* = 816.14) participants were faster than graspable incongruent (*M* = 900.62; *p* < 0.001), big congruent (*M* = 847.48; *p* < 0.05) and big incongruent (*M* = 869.03; *p* < 0.005) confirming the greater facility to elaborate graspable congruent compared to all the other conditions; in addition, they were faster with big incongruent trials than graspable incongruent (*p* < 0.05). **Figure 2** shows all significant results.

## Discussion

In the present experiment we have shown that in a grammatical procedure, the semantic content of grammatical markers affects the speakers’ RTs. In accordance with our hypothesis, our results showed that grammatical violations of classifier-noun combinations produce significantly faster RTs on a grammaticality judgment task when the initial classifier implicitly refers to graspable objects customarily used in hand space, than when the classifier refers to non-graspable objects typically viewed in extrapersonal space. In both conditions, RTs are longer when the classifier-noun pair is incongruent than when they are congruent. However, it was shown that RTs in the graspable classifier incongruent condition are significantly longer compared to the big classifier incongruent condition. Because there is an interaction effect between classifier and grammaticality judgment, it is the classifier rather than the grammaticality judgment that drives the differences in RTs. We have assumed on linguistic grounds, i.e., on the basis of what is known about grammaticalization processes in general and with respect to how these processes have affected nominal classifier stimuli in Chinese in particular, that the content of the stimuli are not only highly abstract mental representations, but also implicitly learned and non-consciously processed. While not being readily accessible to the speakers’ conscious minds, the semantic content of the grammatical units may still be processed in the brain according to embodiment principles. Given the pre-existing research on profound effects on hands’ presence on attention, perception, learning and memory, we argue that these effects for the graspable classifier condition is a result of the special semantics of this grammatical marker, reflecting a readiness for action as well as a difficulty of disengagement effect within the peripersonal space. This demonstrates, in our view, that grammatical categories like the ones under discussion here are grounded.

This is not unlikely because Chinese classifier-noun processing has been shown to trigger ERP effects compatible with semantic as well as syntactic representation. Neuropsychological studies have shown that Chinese numeral classifiers in combination with incongruent nouns evoke an N400 effect, an index of semantic processing or semantic integration, as well as a P600 effect, which is rather associated with syntactic integration (Jiang and Zhou, 2012; Chou et al., 2014). However, which one is primary is still under debate. For example, it appears that while some semantic information is reflected in such studies, not all semantic values trigger the N400 effect; Zhang et al. (2012) report that although congruency mismatch evoke an N400 effect, semantic violations relating to *animacy* in classifier-noun pairs in Chinese does not increase the N400 amplitude. This suggests that at least some conceptual aspects of classifier-noun pairs are not directly involved in conscious semantic integration of classifier and noun. Further evidence indicate that classifier-noun pairs are handled by the brain as a syntactic component; in the same study, the N400 effect was followed by a P600 effect for incongruent animacy-mismatch pairs, but not for incongruent animacy-match classifier-noun pairs. By contrast, Hsu et al. (2014) identifies classifier-noun mismatches as a semantic but not a syntactic violation, since no P600 effect was observed in their results. They used stimuli where the classifier and noun was intercepted by a relative clause, and observed an N400 effect at the appearance of the head noun, suggesting that classifier-noun semantic agreement is retained in memory even at a distance. This duality of semantic-syntactic representation underpins our interpretation of the RT results that the classifiers are grammaticalized in a syntactic automatized procedure, but their processing still depends on semantic representation in the Chinese speakers’ brains.

### Alternative Interpretations: Usage Frequency Effects

Our main claim is that it is the semantic content of the classifier categories that drives the RT effects. However, before reaching a final conclusion, other possible explanations must be ruled out. A well-known contributor to RT effects in language studies is *usage frequency*. If, say, the graspable object classifier with accompanying nouns is far more frequently used than the big object classifier, this could potentially trigger faster RTs in this classifier’s congruent condition, not as a ‘readiness to act,’ but because CL:GRASPABLE.OBJECT-noun combination would be more common and hence easier to retrieve and recognize than CL:BIG.OBJECT-noun combinations.

Two studies consider usage frequencies of the CL:GRASPABLE.OBJECT *bǎ* and CL:BIG.OBJECT *zuò* in various corpora; Zhiqun Xing (2012) gives frequency counts of the 16 most commonly used classifiers in Modern Chinese, selecting out of 178 instances from *A Chinese-English Dictionary of Measure Words [Classifiers]* (Jiao, 2001) classifiers with more than 10,000 occurrences. The frequency counts are based on three grammatical environments where Modern Chinese nominal classifiers are used in the online corpus compiled by the Center of Chinese linguistics, Peking University (2005): on CL occurrences with the numeral *yî* ‘one’, demonstratives *zhé/nà* ‘this/that’, as well as the interrogative words *jǐ* ‘how many’ and *nǎ* ‘which’. The count shows that on the whole, the CL:BIG.OBJECT is in fact used nearly twice as often (24,507 times) as the CL:GRASPABLE.OBJECT (13,531 times). In comparison, the total number of instances of the overall most productive default *gè* classifier stands out by 822, 988 times. See **Table 1**.

**Table 1:** Frequencies of ‘Big’, ‘Grasp’, and default classifiers, taken from a count of the 16 most commonly used (>10,000 occurrences) classifiers in Modern Chinese (Zhiqun Xing, 2012).

Classifier	Semantic function	With numeral	With demonstrative	With question word	Total
zuò big	‘Object with a base’	15,974	8,003	530	24,507
bǎ grasp	‘Hands-on object’	11,440	1,763	328	13,531
gè default	Generic	502,039	268,650	52,299	822,988

In another study, Gao and Malt (2009) include the GRASPABLE.OBJECT classifier *bǎ* and BIG.OBJECT classifier *zuò* in a larger group of 126 specific (‘individual’, in their terminology) classifiers, deemed to be ‘a list of the more commonly recognized classifiers.’ Occurrences and frequency rates were based on language material from both literary and spoken sources: Chinese books, newspapers (People’s Daily, Overseas Edition), dictionaries (Chen et al., 1988), as well as casual conversations between native Chinese speakers in an American university. The selection of classifiers was then subjected to familiarity judgments by six Chinese graduate students (three of them in Beijing). Numbers represent frequencies for the classifier when used in a text corpus of 10 million words. The frequency rates shown here are number of occurrences divided by 1,000. In this material (Gao and Malt, 2009, Appendix A), the grasp classifier *bǎ* received a frequency score of 0.1516 and the big classifier *zuò* a score of 0.2135, indicating again that *zuò* is actually used more frequently than *bǎ* (by comparison, in this material, the highly productive, general classifier *gè* is given a frequency rate of 8.5547). The fact that the general classifier *gè* stands out in terms of productivity is common knowledge among Sinologists. Confer also the correlation of these numbers to our simple Internet search described above. These percentages therefore provide a measure of comparison that the frequencies of our classifiers of interest are correct.

On the basis of these frequency counts, we conclude that the relatively significantly faster RT with the GRASPABLE.OBJECT classifier cannot be attributed to biases caused by frequency effects, since these would rather drive the RT data in the opposite direction and yield shorter RT for congruent trials in the BIG.OBJECT classifier condition than the GRASPABLE.OBJECT classifier condition. In addition, frequency could not explain the significantly longer RT for the incongruent CL: GRASPABLE.OBJECT than for the incongruent CL:BIG.OBJECT conditions.

### RT in Grammaticality Judgment Tasks

Our results showed that incongruent trials had longer RTs than congruent trials, in both conditions. How does this fit with previous studies using this method? Grammaticality judgments in general do not produce unanimous results with respect to typical RTs of grammatical versus ungrammatical constructions. Typically, RTs depend on the **type of grammatical error** made in the stimuli, e.g., errors of omission, agreement or transposition (Blackwell et al., 1996). For agreement errors, typical RTs in Blackwell et al.’s study range from 857 to 643 ms. In some studies, RTs produced for the ungrammatical sentences are overall faster than for the grammatical sentences (Foursha et al., 2006). This, however, depends on the kind of linguistic task given. Post-sentence integration reflects a different type of process than grammaticality judgment of a phrase. Hence, in another study on Spanish by Leeser et al. (2011), which comes closest to the type of grammaticality judgment in our experiment, the gender stimuli used in combinations of noun-adjective was *slower for incongruent* than for congruent noun-adjective combinations. The task and procedure were similar to the ones in our experimental paradigm. The agreement requirements are akin to the grammatical constraints imposed on classifier-noun combinations in that they are *contiguous to the noun* and that there is some sort of *semantic relationship* between modifier and noun (but probably less so for gender agreement). In Spanish, most nouns that are suffixed -a are feminine, and most nouns ending in -o are masculine. The adjective agrees with the noun immediately preceding the adjective by suffixing the same vowels as the noun; for example, cuento corto (masc.) ‘story short’, ungrammatical *cuento corta, and in feminine nouns, pluma roja (fem.) ‘pen red’, ungrammatical *pluma rojo. Twenty-four grammatical/ungrammatical sentence pairs as in (1) were presented where the target phrase was placed in object position.

(1)

a. El hombre limpia una ventana sucia par aver la calle.
The man cleans a_Det-Fem_ window_Noun-Fem_ dirty_Adj-Fem_ to see the street.

b. *El hombre limpia una ventana sucio para ver la calle.
The man cleans a_Det-Fem_ window_Noun-Fem_ dirty_Adj-Masc_ to see the street.

The sentences were revealed in a word-by-word fashion, and a prompt appeared on a separate screen after the sentence with the question “Is the sentence grammatically correct?,” and participants answered “yes” or “no”. *The responses were consistently slower for the incongruent trials*. Thus, the RTs achieved in our experiment reflect what can be expected on this particular type of linguistic task.

### Biological Impact on Linguistic Grounding?

In discussions on the interface between language and thought, the question is often raised (e.g., Schmitt and Zhang, 1998; Zhang and Schmitt, 1998; Saalbach and Imai, 2007; Gao and Malt, 2009; Kuo and Sera, 2009; Huettig et al., 2010; Imai et al., 2010) whether classifier categories have an effect on speakers’ conceptual system, concluding that language structure would shape the way humans think. Our study has pointed out that the reverse effect is a much more likely scenario: that the constraints imposed by our attention systems founded in biology and evolutionary history affect our general cognitive processes, which again determine certain aspects of linguistic structure in favor of grounding abstract grammatical categories directly in relation to the modalities that were active in the acquisition process. From cognitive psychology it is known that as children’s brains’ motoric systems develop, new action systems emerge in parallel. In this process, they generate new abilities such as attending to objects and exploring them, learning to control events and their potential outcomes. This is in accordance with the facts outlined in the present study that some features of language may be shaped by constraints outside of language structure itself, even in the case of implicitly acquired grammatical categories. We see this reflected in language learning: although children master nominal classifier expressions rather early, the acquisition of the semantic system underlying the partitions is much slower, and may not fully resemble the adult system even at the age of nine (Aikhenvald, 2000). Interestingly, which classifiers are acquired at what ages seems not to be entirely random. In Chinese, only the three dimensional small (i.e., graspable) object classifiers are mastered up to the age of four in addition to the human classifiers (Erbaugh, 1986). We know that at 2 years of age, only gross motor skills are fully developed in children; they can for example jump and walk. A similar study on first language acquisition of Japanese nominal classifiers parallels the idea that categories for small objects are acquired early, but adds the dimension of fine motor skills. Yamamoto and Keil (2000) reports that while the power-grasp classifier -*ko* for small, graspable objects is the first shape-specific classifier to be learnt around the age of two, the pinch-grip classifier used of tiny objects like grains of rice, -*tsubu*, is acquired much later, around the age of five (cf. Matsumoto, 1985a,b, 1987, citing Okubo, 1967 and Murata, 1983). Gross motor skills develop prior to fine motor skills, and not until the age of three can a child use scissors for cutting or run on her toes. This development of linguistic categories therefore **parallels motoric development** in children. In her study on parallels between the acquisition and historical development of Chinese nominal classifiers, Erbaugh also argues that **sensorimotor reinforcement** is vital to early classifier use.

Language learning suggests that building object knowledge and the corresponding concepts and categories begins with physical interactions with concrete objects in the external world. Chinese children’s acquisition of classifiers is designated by distinct stages that reflect going from specific item to concrete objects to generalizations over types. In study Erbaugh’s (1986), the use of specialized semantic classifiers on the whole was rare and developed slowly in children in the age span 1.10 (1 year and 10 months) to 3.10. At this stage (stage 1) they associated specialized classifiers lexically to a specific referent. (This pattern is also attested in the historical development of the classifier system from ancient to modern Chinese). When classifier use started developing (stage 2), children generalized from a prototype while they also overgeneralized and used the classifier in *incorrect but plausible ways*, e.g., the extension of bǎ (把) ‘graspable objects’ incorrectly to *fish* [where the correct classifier is wěi (尾)]. For our purposes it is interesting to note that one of the two most frequently generalized classifiers in child language data refer to small size, i.e., graspable objects. Furthermore, in this stage, which occurred after age 2.6, the children ‘used classifiers exclusively for concrete objects which they had handled.’ The two children aged 2.10 and 2.6 only used classifiers that referred to movable, concrete objects, and the same was the case in 63 out of 68 classifiers used by the older child at 3.10. Erbaugh points out that this is in accordance with psychological principles of early learning, the Piagetian *action schema*. In support of our assumption that grammatical categories can be biologically grounded, Erbaugh (1986) reasons that classifiers evidently reveal *already-emerging conceptual categories* rather than pre-shaping them.

In what way could these concepts be ‘already-emerging’ prior to language acquisition? And how does the brain derive unitary representations of external space across different modalities? The existence of multisensory neurons and receptive fields (RFs) constitute a **neuroscientific explanation** to near space attention as well as to object size. Given the presumably more complex process of integrating two modalities, vision and touch, why should graspability induce shorter RTs? The answer might be the existence of specialized bimodal visuotactile neurons in certain brain areas, so-called *multisensory neurons*.

Knowledge of graspable vs. large size objects depends on multimodal integration in the brain. A large body of **neuropsychological data** supports a subdivision of space whose definition depends solely on the potential graspability of objects. This subdivision separates space into two domains: the near or *peripersonal* space, and the far or *extrapersonal* space (Brozzoli et al., 2012; Nemmi et al., 2013) – corresponding to within and beyond arm’s reach, quite literally defined. This space is malleable and will temporarily shrink if access to peripersonal space is reduced, e.g., by placing a panel between a participant and the graspable object (single cell recordings of monkey: Caggiano et al., 2009), or increase, when participants are provided with a tool; neurons in post-central gyrus and intraparietal sulcus began to display visual responses after monkeys were trained to use a rake to reach food pellets (Iriki et al., 1996). Processing of near and far space is believed to rely on two different cortical visual processing streams (Ungerleider and Mishkin, 1982; Goodale and Milner, 1992). The different types of visual processing have **implications for attention**. Normal as well as pathological asymmetries of visuospatial attention may differ depending on the distance of stimulus presentation (Shelton et al., 1990; Halligan and Marshall, 1991; McCourt and Garlinghouse, 2000; Varnava et al., 2002; Committeri et al., 2007). Heber (2010) concludes that differences in attentional asymmetries with respect to depth suggest dissociable neural mechanisms for visual attentional processing in near and far space. Intersecting the near-far space division one finds two separate but overlapping spatial attention systems which underlie human behavior, suggested to reflect a phylogenetically old mechanism developed for information processing to handle further potential action (Chica et al., 2013). One is oriented endogenously to stimuli that are relevant for the task at hand and is directed at will or expectancy of where stimuli appear; the other is oriented exogenously and serves to avoid danger of unexpected happenings. Typical triggers of exogenous attention would be saliency markers like luminance changes, onsets, or moving stimuli. The definition of peripersonal space actually originates from studies using single-cell recordings in the macaque brain (Brozzoli et al., 2012). These recordings demonstrated the existence of multisensory neurons in several areas in the cat and monkey brain: the putamen, superior colliculus, ventral and dorsal premotor cortex (BA6), and parietal areas 7b and the ventral intraparietal sulcus (Rizzolatti et al., 1987; Fogassi et al., 1999; Graziano, 2001). Cells in these areas that are responsive to active stimuli on the animal’s arm and hand have RFs for the region of space close to the animal’s arm. The visual RFs of these neurons follow the hand around when the experimenter places the arm in different postures (Graziano and Botvinick, 2002). In other words, these bimodal neuron populations encode space not according to retinotopic or head positions, but on the basis of a hand-centered coordinate system (Rizzolatti et al., 1997). If the hand is moved, the neurons will instead respond to the hand’s new position (Reed et al., 2006). RFs are independent of gaze direction (Gentilucci et al., 1983; Fogassi et al., 1992), and visually evoked responses of these neurons are instead modulated by the distance between the visual object and the tactile RF (Brozzoli et al., 2012). The effect of this modulation is that visual information can be coded that is dependent on the body part containing the tactile RF; for example, large RFs in premotor cortex of the monkey brain containing tactile neurons discharge in response to visual stimuli.

Notably, special RF areas are particularly responsive to *object size*; in other words, **object affordances** are encoded directly in the individual’s brain. Visuotactile neurons in rostral subregion F5 of area 6, although less numerous than in the F4 RF neurons, are driven by stimuli size more than by closeness to body (Rizzolatti and Gentilucci, 1988; Rizzolatti et al., 1988). The motor properties of premotor and parietal areas, as well as the interconnectivity of the premotor cortex to the primary motor cortex and to the spinal cord, suggest that the role of these structures are not just visual-perceptual, but rather part of a **perception-action interface**, where they subserve the body parts’ approaching movements toward an object (Gardner et al., 2007; Brozzoli et al., 2012), and are part of the dorsal stream for grasping. Similar multisensory representations of peripersonal space in humans are supported by numerous behavioral (e.g., Spence et al., 2004; Lloyd et al., 2006; Makin et al., 2007), and neuroimaging studies (Lloyd et al., 2003), indicating that the same network of neural structures is involved in the multisensory representation in primates – the VIP-F4 circuit (Rizzolatti et al., 2002).

These multisensory spatial representations can be impaired in humans as well as in monkeys following selective brain damage (Spence et al., 2008). Importantly, damage to the relevant visuotactile and parietal regions can cause *attention deficits* in the form of hemineglect in monkeys, restricted to peripersonal space (Rizzolatti et al., 1983). Crucially, if peripersonal space is represented in a similar fashion in several species, there is reason to believe that the special status of the area immediately surrounding the body is an evolutionary trait, and not just a result of each individual’s learning process. In other words, concepts implying this space are **grounded by biology**. This points to the idea that much of the neural hardware involved in language is not specific to it (Christiansen, 2013).

However, one faculty might not preclude the other. Just as humans are thought to be neurally equipped at birth to develop language skills, they may have an innate biological predisposition for linking vision and action. The fact that fast instinctive physical reactions (e.g., shielding with hand, blinking with eye) is linked to the (anticipatory) avoidance of rapidly approaching objects into the visual field in near space (Fogassi et al., 1996; Cooke and Graziano, 2004; Graziano and Cooke, 2006) provides additional support for an evolutionarily determined co-development of vision and motor action. It is important here to stress that voluntary object-oriented actions, such as would be the neurocognitive basis for the graspable object classifier, *also can be anticipatory* (Rizzolatti et al., 1981a,b, 1997; Gardner et al., 2007).

In total, these studies indicate that not only are the mechanisms underlying perception, attention, and memory dependent on the relationship between perceivers and their environment, they also demonstrate that this interdependence relates to the individual’s planned motor action within action space. If these psychological and biological facts are continually shown to impact on the mental representations of grammars, the connection between these higher-order, abstract grammatical markers to biology and general cognition can successfully be explained in terms of linguistic grounding.

### Grounding Abstract Sensorimotor Referencing Categories; Implications for Theory

A discussion still goes on about the adequacy of embodied cognition to account for *abstract concepts*. Failure to address this question implies failure to vouch for embodied cognition as a general theory for language, since abstract concepts will then only be satisfactorily accounted for in amodal systems. Various neuroscientific models of conceptual representation have been proposed in varying degrees of embodiment, the representatives of which have tended to guard their perspective vigilantly against the opposing standpoints (for a review, see Meteyard et al., 2012). The new trend in the embodiment debate, however, is to propose that models should show how and to what extent embodied concrete and abstract concepts are *combined* in language use and grammar, thus positing a theoretical and pragmatic *opposition* between ‘abstract’ and ‘grounded’ within the same linguistic model (see, for instance Vigliocco et al., 2009; Arbib et al., 2014; Zwaan, 2014). Underlying these attempts is the assumption that abstract and embodied concepts are somehow mutually exclusive. For example, Zwaan (2014) poses an opposition between ‘abstract’ and ‘grounded’ in saying that ‘Accepting that we need both abstract and grounded symbols leads to a new set of questions. When do we need which and how do they interact?’ The same contrast is inherently assumed in Moseley and Pulvermüller (2014) when they discuss whether the brain distinguishes primarily between grammatical categories (nouns and verbs) or is topographically organized according to sensorimotor semantic classes in amodal vs. embodied theories.

Our investigation, by contrast, demonstrates that there need be no notional nor empirical contradiction between ‘sensorimotor’ and ‘abstract’, since abstraction is a process of the mind that has less to do with physical tangibility than with the typical summary representation that *any concept* is based on, irrespective of experiential basis or placement along the vertical axis in taxonomic-linguistic and grammatical hierarchies. This is evident by the stimuli used in our experiment; nominal classifiers that generalize over sensorimotor interaction abstractly representing sensorimotor affordances in grammatical classes. Pulvermüller (2013) discusses four separate mechanisms of establishing ‘symbolic meaning’, two of which refer to the ‘abstraction mechanisms for generalizing over a range of instances of semantic meaning’ and ‘referential semantics’, ‘which establishes links between symbols and their objects and actions they are used to speak about.’ In our investigation, however, we have shown that there is no contradiction between ‘referential semantics’ and ‘abstract semantics’, since classifiers are deictic, abstract grammatical elements.

The difference between abstract and concrete is better explained in terms of **specificity**. The concrete-abstract scale is typically inversely related to the number of members in each category, i.e., the less properties are necessary to describe the category, the more abstract it is and the more members fit into the category. We relate here to the original sense of the word ‘abstract’ as it was derived from Latin abstractus, perfect passive participle of abstrahô, formed from abs- (“away”) + trahô (“draw”), pointing to the mind’s ability to extract from single referents and generalize over several experiential instances in the process of forming ever more general concepts. That is, a concept is not formed by recalling one concrete instance but by comprising all previous encounters in a common mental representation, be that encounters with similar objects and situations in the real world in the formation of, e.g., noun concepts, or generalizations over a class of lexical units such as the nouns themselves. Since nominal classifiers generalize over nouns, they are in fact synchronically *abstractions over abstractions*. This process requires a mind’s ability to organize within a hierarchical structure, and when a word changes into a grammatical marker abstraction happens along this vertical axis. One of the successful traits of human language as an efficient communication device is the development of mental grammars. The ability to abstract is a precondition for developing grammars. Grammatical morphemes come about as a result of grammaticalization processes, in which abstraction equals lessening the semantic content while increasing their general value of application. The specific mechanism is the above outlined capability that the brain has to recognize similarities across different concrete experiences and extract a mental representation generalization, which are applicable to and comprises all former encounters.

While advocating a basic division between ‘abstract’ and ‘concrete-grounded’, Zwaan (2014) also allows for embodied representation of low specificity concepts: ‘There were 50 red roses in the vase,’ which specifies the exact number of roses, and ‘Several people entered the house,’ which underspecifies number, gender and age of the people entering. While both sentences may be mentally represented and grounded in sensory and motor experiences, detailed representation is not a prerequisite for a concept to be grounded. Our experiment confirms this supposition. In the present investigation we have shown that ‘abstract’ need not be mutually exclusive of ‘grounded’.

Conceptualisation is the founding mechanism and key success factor of language, the types of which differ only in **complexity** and **type of experiential bases**. In principle, there is no type of experiential basis that language could not turn into an abstract concept, and sensorimotor experiences are no exception. The fact that referencing intangible experiences and generalizing over multiple encounters are fundamentally different processes is evident by the fact that languages also conceptually represent generalizations over intangible referents, for example emotions; Vietnamese has two nominal classifiers for emotions: niềm ‘sentiment’ defines a short-term sentiment that arises in a certain situation (*grief, rage, joy*) and nỗi ‘feeling’, which defines a sentiment that lasts for a while (*the sadness of a refugee, aﬄiction, despair*; Trần, online). The folk etymology extension of ‘abstract’ to comprehend intangible referents might have arisen out of the realization that generalizing over multiple encounters of items in effect amounts to each of the items being intangible.

The confusion may also be due to the fact that languages may employ **several mechanisms simultaneously**. In metaphorical extensions, concrete sensorimotor experiences often serve as a basis for highlighting features of mental, emotional, social interaction or grammatical phenomena. Such conceptualisations use feature extraction in *exactly the same way* as in feature extraction of encounters with concrete objects and situations that form nouns and verbs, but in addition represent intangible experiential domains. For example, in Hausa grammar (West Africa), the same morpheme -r is used to mark physical causation and mental coercion. For example, adding the -r suffix to the verb *zaunàa* ‘to sit (intransitive)’ creates *zaunar* ‘to seat (somebody)’; likewise, in mental transfers like *kooyar* ‘to teach’ are derived from a base form *kòoyaa* ‘to learn’ (Lobben, 2010). In other words, turning physical situations into the mental transfers of social situations is one of the basic building blocks of language. In such grammaticalization processes, a word may be selected for either one or several of paths of transfer and differential features of the basic word may be highlighted, all of which are based in a basic, often sensorimotor experience. As we saw for Chinese, the previously existing, physical interaction verb *ba ‘to grasp’ developed into denoting the abstract knowledge of affordances of graspable objects (the classifier), a purposive marker signifying *intent*, or the metaphorical-abstract understanding of *acting on an object* (the transitivity marker). Whether or not the two latter instances of *ba*, grammatical procedures that do not necessarily imply interaction with objects in the acquisition process, are also grounded, remains an open question.

### Grammaticality Judgments Reflect Immediate and Unattended Conceptual Processing

Critics of embodied theories have suggested that modal activations are due to mental imagery, i.e., to *post-comprehension processes*. The physiological effects in modal areas could thus be caused by a ‘post-understanding inference’ (Mahon and Caramazza, 2008; Papeo and Caramazza, 2013, 2014). Pulvermüller (2012) and Shtyrov et al. (2014) point out that *immediacy*, *functional relevance* and *automaticity* each suggests that modal activations are intrinsic rather than epiphenomenal to conceptual processing. Viewed against research on peripersonal hand space, differential RTs rather suggest immediacy, functional relevance and automaticity of visuomotor effects on attention in hand space for abstract grammatical categories. They are immediate because they appear to be directly grounded in biological processes (cf. discussion above); they are functionally relevant because the visuomotor classifier evidently activates sensorimotor readiness for action more than the visual modality classifier; and they are automatic because grammatical concepts are not attended to consciously. Our results are therefore a valid contribution to the discussion on the necessity of embodiment in conceptual representations in the brain.

### Future Directions

While studies on grounding by sensorimotor interaction of *lexical-level* linguistic concepts are abundant and keep being published (Barsalou, 2008; Moseley et al., 2011; Innocenti et al., 2014; Moseley and Pulvermüller, 2014), studies using abstract *grammatical* stimuli are only starting to appear (e.g., Tettamanti et al., 2008; Bergen and Wheeler, 2010; Capelle et al., 2010). We would encourage more research on grammatical morphemes and the semantics of syntactic structures (Goldberg, 2006). We would also encourage proper linguistic analyses to be carried out on stimuli. A practice in the field still prevails that conflates as ‘abstract’ the two fundamentally different phenomena: intangible referent and generalizing over variation. Many of the studies within the embodied cognition framework relating to abstract concepts deal with internal state concepts such as emotions (e.g., Moseley et al., 2011). In such studies, abstract equals internal state, and although we shall not *a priori* exclude the possibility that internal state experiences and generalizations can be associated at word level semantics (e.g., complex situational with emotional connotations as in ‘peace’, or internal-external experience based concepts such as ‘beauty’), it is important to notionally separate these in research procedures.

## Conclusion

Our interest in the present investigation was in finding out whether what is known about attention and neurophysiological bias for graspable objects in hand space had any effect on the processing of abstract, grammatical categories.

It is known that while endogenous attention is a top–down process developed for immediate action, exogenous attention is a reactive process driven by bottom–up perceptive stimuli for which the individual is not already prepared. Each attention system modulates the individual’s performance, accommodating intentional goals and environmental circumstances. This is in line with the typical characteristics of an individual’s peripersonal space, a region of space that affords considerations of how to prepare appropriate course of actions in near space corresponding to top–down mental processes. It also explains why actions in near space are characterized by a special immediacy. Faster RTs to the nominal classifier for graspable objects in Chinese are likely a reflection of this immediacy: action is driven by endogenous attention and intention as a result of previous experience with handheld objects. The same real-world experience showed a slow disengagement effect for the graspable object classifier, reflecting a person’s uninclined disposition to alter an attention focus that is invested in near space. The nominal classifier referring to knowledge about big sized objects, by contrast, although salient by virtue of their largeness, did not spur similar effects. When considering the reported results against the backdrop of existing literature on hand-related physiological and cognitive processes we hold it to be likely that the graspable nominal classifier is differentially grounded from the big nominal classifier in Chinese.

## Conflict of Interest Statement

The authors declare that the research was conducted in the absence of any commercial or financial relationships that could be construed as a potential conflict of interest.

## Footnotes

1 In an Internet search we found more than 18, 2 million occurrences of bǎ combined with the number one (一把). In comparison, the non-general, highly specialized pǐ (匹) classifier used only of horses produced only 1,25 million instances. The big object zuò (座) classifier gave 46,5 million, and the most productive default gè (座) classifier yielded 614 million instances.
