# Supplementary material for: Grounding grammatical categories: attention bias in hand space influences grammatical congruency judgment of Chinese nominal classifiers
Source: Front Psychol. 2015 Aug 27;6:1299. doi: 10.3389/fpsyg.2015.01299 (PMC4550751; doi:10.3389/fpsyg.2015.01299)
Supplement: Supplementary file 1 [file Data_Sheet_1.DOCX]

**Appendix**

**Table 1.** Nouns used in the congruent graspable objects classifier condition (一把)

Example: 一把 ‘one graspable-object.CL’ 锤子 ‘hammer’

| **Noun** | **Meaning** |
| --- | --- |
| 夹钳 | A (pair of) tongs |
| 镰刀 | A scythe, sickle |
| 卷尺 | A tape measure, tape rule |
| 吉他 | A guitar |
| 锤子 | A hammer |
| 钥匙 | A key |
| 叉子 | A fork |
| 二胡 | A Chinese two-string fiddle |
| 锄头 | A hoe |
| 手枪 | A pistol |
| 锹 | A spade |
| 钳子 | A (pair of) pliers, pincers, tongs, forceps, vise, clamp, claw |
| 锥子 | An auger, an awl |
| 铲子 | A shovel, spade, trowel, spatula (kitchen utensil) |
| 宝剑 | A double-edged sword |
| 镰刀 | A sickle, curved blade to cut grain |
| 扳子 | A spanner, wrench |
| 墩布 | A swab, mop |
| 铁锨 | An iron shovel, spade |
| 改锥 | A screwdriver |
| 算盘 | An abacus |
| 扫帚 | A broom |
| 香蕉 | A banana |
| 刀 | A knife |
| 剪子 | A (pair of) clippers, scissors, shears |
| 菜刀 | A vegetable/kitchen knife, cleaver |
| 斧子 | An axe, hatchet |
| 牙刷 | A toothbrush |
| 火炬 | A torch |
| 梳子 | A comb |
| 雨伞 | An umbrella |
| 勺子 | A spoon, ladle |
| 扇子 | A (folding) fan |
| 剪刀 | A (pair of) scissors |
| 长矛 | A pike, a lance |
| 斧头 | A hatchet |
| 笤帚 | A whisk broom, small broom |
| 椅子 | A chair |
| 刷子 | A brush, scrub |
| 剑 | A sword |

**Table 2.** Nouns used in the incongruent graspable objects classifier condition (一把)

| **Noun** | **Meaning** |
| --- | --- |
| 林荫大道 | A boulevard, tree-lined avenue |
| 鲸鱼 | A whale |
| 渔船 | A fishing boat |
| 山脉 | A mountain range |
| 胡同 | A lane, alley |
| 大路 | An avenue |
| 渠 | A big stream/canal, ditch/drain |
| 河流 | A (flowing) river |
| 马路 | A street, road |
| 坝 | A dam, dike, embankment |
| 铁路 | A railroad, railway |
| 街道 | A street; subdistrict, residential district |
| 公路 | A highway, road |
| 街 | A street |
| 龙 | A dragon |
| 船 | A boat, vessel, ship |
| 河 | A river |
| 牛 | An ox, cow, bull |
| 江 | A river |
| 沟 | A ditch |

**Table 3.** Nouns used in the congruent big objects classifier condition (一座)

Example: 一座 ‘one big.CL’ 山 ‘mountain’

| **Noun** | **Meaning** |
| --- | --- |
| 摩天大厦 | A skyscraper |
| 办公楼 | An office building |
| 山 | A mountain, hill |
| 楼房 | A building of two or more stories |
| 大楼 | A large building |
| 坟 | A tomb, grave |
| 寺院 | A cloister, monastery, temple |
| 厂房 | A factory building |
| 百货大楼 | A department store |
| 公园 | A park for public recreation |
| 水库 | A reservoir |
| 高楼 | A high building, multistory building, skyscraper |
| 城堡 | A castle |
| 别墅 | A villa |
| 房子 | A house, building (single or two story), apartment, room |
| 冰山 | An iceberg |
| 宫殿 | A palace |
| 村庄 | A village, hamlet |
| 花园 | A garden |
| 工厂 | A factory |
| 避风港 | A haven, refuge, harbor |
| 岛 | An island |
| 高塔 | A tower |
| 大厦 | A large building, mansion, edifice |
| 铜像 | A bronze statue |
| 堡寨 | A fort, fortress |
| 城池 | A city |
| 塔 | A tower, pagoda, minaret, stupa |
| 大使馆 | An embassy |
| 宿舍楼 | A dormitory building |
| 摩天楼 | A skyscraper |
| 灯塔 | A lighthouse |
| 铜像 | A bronze statue |
| 庙 | Temple, ancestral shrine |
| 桥 | A bridge, structure, architecture, composition, makeup |
| 剧院 | A theater |
| 公寓楼 | An apartment building |
| 住宅楼 | A residential building |
| 荒岛 | A barren or uninhabited island |
| 体育场 | A stadium |

**Table 4.** Nouns used in the *incongruent big* objects classifier condition (一座)

| **Noun** | **Meaning** |
| --- | --- |
| 小麦 | A grain of oats |
| 钻石 | A diamond |
| 小米 | A grain of millet |
| 豌豆 | A pea (pisum sativum) |
| 沙子 | A grain of sand; grit |
| 糖果 | A candy |
| 种子 | A seed |
| 麦子 | A grain |
| 珠子 | A bead |
| 小麦 | A (grain of) wheat |
| 胶囊 | A medical/pharmaceutical capsule, caplet |
| 子弹 | A bullet |
| 葡萄 | A grape |
| 冰雹 | A hail, hailstone |
| 灰尘 | A mote |
| 龙眼 | A dragon eye fruit/longan |
| 药丸 | A pill |
| 尘土 | An earth-/dust lump (small) |
| 弹子 | A slingshot pellet, playing marble |
| 玉米 | A (grain of) corn, maize |
